# Elevated gut microbiota metabolite bile acids confer protective effects on clinical prognosis in ischemic stroke patients

**DOI:** 10.3389/fnins.2024.1388748

**Published:** 2024-04-08

**Authors:** Zhaobin Wang, Jing Li, Yingxin Xu, Ye Liu, Zhe Zhang, Qin Xu, Jinxi Lin, Yong Jiang, Yongjun Wang, Jing Jing, Anxin Wang, Xia Meng

**Affiliations:** ^1^Affiliated Hospital of Hebei University, Baoding, China; ^2^Clinical Medical College, Hebei University, Baoding, China; ^3^Department of Neurology, Beijing Tiantan Hospital, Capital Medical University, Beijing, China; ^4^Puyang Oilfield General Hospital, Puyang, China; ^5^China National Clinical Research Center for Neurological Diseases, Beijing Tiantan Hospital, Capital Medical University, Beijing, China; ^6^Department of Neurology, Beijing Daxing District People's Hospital, Beijing, China; ^7^Department of Anaesthesiology, Beijing Obstetrics and Gynecology Hospital, Capital Medical University, Beijing, China

**Keywords:** gut microbiota metabolite, bile acids, gut-brain axis, poor functional outcome, stroke

## Abstract

**Background:**

There is evidence of an association between the gut microbiota and progression of stroke. However, the relationship between gut microbial metabolites, specifically bile acids (BAs), and post-ischemic stroke disability and poor functional outcomes remains unexplored.

**Methods:**

Patients with acute ischemic stroke (AIS) or transient ischemic attack (TIA) in the Third China National Stroke Registry were grouped according to total bile acid (TBA) quartile on admission. Association of TBA with disability and poor functional outcomes were evaluated using logistic regression models and restricted cubic splines.

**Results:**

Data for 9,536 patients were included. After adjusting for confounders, the risks of disability and poor functional outcomes were significantly lower in the highest TBA quartile than in the lowest TBA quartile at the 3-month follow-up, with respective odds ratios (ORs) of 0.65 (95% confidence interval [CI] 0.55–0.78; *p* < 0.001) and 0.66 (95% CI 0.55–0.78, *p* < 0.001). Each standard deviation increase in the TBA level reduced the risks of disability and poor functioning outcomes by 10% (adjusted ORs 0.9 [95% CI 0.83–0.98; *p* = 0.01] and 0.9 [95% CI 0.83–0.97; *p* < 0.001], respectively). This association remained similar at the 1-year follow-up. After stratification by TOAST subtype, the risk of disability or a poor functional outcome in patients with the large-artery atherosclerosis or “other” subtype was significantly lower in the highest quartile than in the lowest quartile (*p* < 0.05).

**Conclusion:**

Serum TBA is an independent risk factor for disability and poor functional outcomes after AIS or TIA, and exerts a protective effects on brain.

## Introduction

The intestinal microbiota has become as a central regulator of immune responses within the gut-brain axis. Dysbiosis of the gut microbiota can lead to communication with the central nervous system via microbial metabolites, neural pathways, and endocrine and immune signaling cascades, thereby contributing to various neurological diseases and neurodegenerative disorders, including stroke, multiple sclerosis, Alzheimer’s disease, and Parkinson’s disease (Cryan et al., 2020; Agirman et al., 2021; Margolis et al., 2021; Singh et al., 2022). Bile acids (BAs) are metabolite of intestinal microorganisms and play a crucial role in modulating gut microbiota balance, making them key players in the gut-brain axis (Wahlström et al., 2016; Cai J. W. et al., 2022). BAs are metabolized by gut microbiota into secondary bile acids, such as ursodeoxycholic acid and tauroursodeoxycholic acid (TUDCA). These acids cross the blood–brain barrier, reducing cell apoptosis, reactive oxygen species (ROS), nitric oxide (NO), tumor necrosis factor (TNF)-α, and interleukin (IL)-1β production, exhibiting anti-apoptotic, antioxidant, and anti-inflammatory properties (Huang F. et al., 2022; Khalaf et al., 2022). Abnormal BA metabolism leads to gut microbiota dysbiosis, resulting in immune cell abnormalities, such as T cells, B cells, lymphocytes, and white blood cell interleukins, impacting the prognosis of stroke patients (Arya and Hu, 2018). Moreover, BAs also influence lipid metabolism and are central in regulating atherosclerosis through interactions with the intestinal microbiota (Jonsson and Bäckhed, 2017; Cao et al., 2023). Studies have shown that decreased BA excretion is associated with atherosclerosis and an elevated risk of coronary artery disease (CAD) (Charach et al., 2020). Additionally, a lower total bile acid (TBA) level has been independently associated with the severity of coronary lesions in patients with confirmed or suspected CAD (Li et al., 2020). Epidemiological investigations have revealed a negative association between serum TBA levels and mortality in patients with acute ischemic stroke (AIS) (Huang L. L. et al., 2022). In the context of hemorrhagic stroke, an elevated serum TBA level has shown potential associations with reduced hematoma size at the time of intracerebral hemorrhage and a better prognosis (Wang et al., 2018). These findings suggest that the BA levels may serve as prognostic indicator in stroke patients. However, no study to date has examined the relationship between serum TBA levels and disability or poor functional outcomes in patients with AIS or transient ischemic attack (TIA). Furthermore, the associations between TBA levels and disability as well as poor functional outcomes have not been explored based on stroke etiology.

In this study, we investigate the relationship between the baseline serum TBA level and the clinical prognosis in patients with AIS or TIA as well as the significance of TBA in the different Trial of Org 10,172 in Acute Stroke Treatment (TOAST) subtypes using data from the Third National Stroke Registry (CNSR-III).

## Methods

### Participants

CNSR-III was a large-scale prospective cohort study conducted in China, spanning 201 hospitals across 22 provinces and four municipalities. 15,166 patients were consecutively recruited from August 2015 to March 2018 and provided demographic information, clinical data, radiological images, and laboratory results for prognostic factor identification. The study’s detailed design and information have been previously published (Wang et al., 2019). Inclusion criteria comprised a diagnosis of AIS or TIA, age 18 years or older, and diagnosis within 7 days of the index event of ischemic stroke or TIA. Patients who did not have TBA measurement within 24 h of admission were excluded (n = 4,913). In addition, patients with liver disease (n = 100), kidney disease (n = 131), gastrointestinal disease (n = 218) before or on admission were excluded, or missing BA values or mRS scores at the 1-year or 3-month follow-up (Supplementary Figure S1). The study was approved by the ethics committee at Beijing Tiantan Hospital (approval number KY2015-001-01), and informed consent was obtained from all participants or their legally authorized representatives.

### Collection of baseline data

After admission, a trained neurologist assessed all study participants, recording baseline data, including age, sex, body mass index, smoking and alcohol consumption habits, medical history (including diabetes, hypertension, dyslipidemia, coronary heart disease, and previous stroke), and drug exposure during hospitalization (antiplatelet agents, anticoagulants, and lipid-lowering therapy). National Institutes of Health Stroke Scale (NIHSS) scores were documented at admission, and pre-stroke modified Rankin Scale (mRS) scores were recorded. Fasting blood samples were collected within 24 h of admission, with serum TBA, alanine aminotransferase, and aspartate aminotransferase levels tested using standard methods at regional laboratories. Blood samples were transported through a cold chain to the central laboratory at Beijing Tiantan Hospital. Central laboratory tests included measurements of total cholesterol, high-density lipoprotein, low-density lipoprotein, triglycerides, and high-sensitivity C-reactive protein (hs-CRP). The estimated glomerular filtration rate (eGFR) was calculated using the Chronic Kidney Disease Epidemiology Collaboration equations, adjusted for the Chinese population with an adjusted coefficient of 1.1 (Teo et al., 2011).

All imaging data were collected in DICOM format, analyzed by two experienced neurologists, and categorized by TOAST etiology. Patients with stroke of other determined etiology were grouped with those with stroke of undetermined etiology, collectively termed “Others.” Patients were classified into four subtypes: large-artery atherosclerosis (LAA), cardioembolism, small-vessel occlusion, and Others.

### Follow-up and assessment of clinical outcomes

Trained research coordinators, blinded to participants’ baseline characteristics, conducted face-to-face interviews at the 3-month follow-up and telephone interviews at the 1-year follow-up after symptom onset to ascertain clinical outcomes. Clinical outcomes were defined as disability and poor functional outcomes at the 3-month and 1-year follow-ups. The mRS score, ranging from 0 (no symptoms) to 6 (death), was used to assess functional status. A poor functional outcome was defined as an mRS score of 3–6, while major disability was defined as an mRS score of 3–5.

### Statistical analysis

Patients were categorized into four groups (Q1, <2.1umol/L; Q2, 2.1–3.7 umol/L; Q3, 3.7–6.1 umol/L; Q4, ≥6.1 umol/L) based on quartiles of TBA levels on admission. Q1 was designated as low, and Q4 as high. Continuous variables underwent normality analysis using the Kolmogorov–Smirnov test. They were summarized as the mean (standard deviation) through one-way ANOVA when all four groups exhibited normal distribution, or as the median (interquartile range) using the Kruskal-Wallis test if one group deviated from normality. Categorical variables were compared using either the Chi-square test or Fisher’s exact probability method. Logistic regression models were used to evaluate the associations between TBA levels and disability, as well as poor functional outcomes, at 3 months and 1 year. Odds ratios (ORs) with 95% confidence intervals (CIs) were calculated for serum TBA levels when categorized by quartile (using the lowest quartile as the reference) and when treated as a continuous variable (per standard deviation [SD] increase).

We employed three adjusted models. Model 1 was adjusted for age and sex. Model 2 was further adjusted for body mass index, current smoking, alcohol consumption, pre-stroke mRS score, TOAST classification, hypertension, diabetes, dyslipidemia, coronary heart disease, and previous stroke. Model 3 was further adjusted for antiplatelet agents, anticoagulant agents, eGFR, and hs-CRP. To visualize potential nonlinear associations of serum TBA with disability and poor functional outcomes, we constructed restricted cubic splines with three knots at the 10th, 50th, and 90th percentiles. Stratified analyses were performed in TOAST subgroups.

All statistical analyses were performed using SAS version 9.4 (SAS Institute Inc., Cary, NC, United States) and R software version 4.1.3 (R Foundation for Statistical Computing, Vienna, Austria). A two-tailed *p*-value <0.05 was considered statistically significant.

## Results

### Baseline characteristics

The final analysis encompassed 9,536 patients. Supplementary Table S1 demonstrates a balanced distribution of baseline characteristics between the included and excluded patients. The patients were assigned into groups Q1 (<2.1umol/L, *n* = 2,243), Q2 (2.1–3.7 umol/L, *n* = 2,457), Q3 (3.7–6.1 umol/L, *n* = 2,428) and Q4 (≥6.1 umol/L, *n* = 2,408) according to the quartiles of fasting serum TBA concentrations on admission. Table 1 summarizes the baseline characteristics of the included patients, revealing a mean age of 62.2 (11.3) years, with 67.8% being male. Current smokers accounted for 31.9% of the patients, while 14.1% were heavy alcohol consumers. The median NIHSS score was 3 (1.0, 6.0), indicating a mild stroke. Patients in the highest serum TBA quartile were more frequently male, slightly older on average, less have diabetes, and more have hyperlipidemia. Conversely, those in the lowest TBA quartile had more history of coronary heart disease. The hs-CRP levels showed a decreasing trend with increasing serum TBA levels, while aspartate aminotransferase levels exhibited the opposite pattern. The TOAST classifications of LAA and Undetermined etiology were relatively high in all TBA quartiles (Table 1).

**Table 1:** Baseline characteristics by total bile acid quartile.

Characteristics	Quartiles of TBA					
	Overall	Q1 (<2.1)	Q2 (2.1–3.7)	Q3 (3.7–6.1)	Q4 (≥6.1)	*p*-value
n	9,536	2,243	2,457	2,428	2,408	
Age (years), mean (SD)	62.2 (11.3)	61.1 (11.7)	61.4 (11.1)	62.5 (11.3)	63.9 (10.7)	<0.001
Men, n (%)	6,469 (67.8)	1,409 (62.8)	1,679 (68.3)	1,694 (69.8)	1,687 (70.1)	<0.001
Body mass index (kg/m^2^), mean (SD)	24.6 (3.3)	24.7 (3.5)	24.7 (3.2)	24.7 (3.3)	24.5 (3.2)	0.487
Current smoking, n (%)	3,045 (31.9)	702 (31.3)	829 (33.7)	759 (31.3)	755 (31.4)	0.173
Heavy drinking, n (%)	1,347 (14.1)	283 (12.6)	366 (14.9)	343 (14.1)	355 (14.7)	0.104
Pre-stroke mRS score, median (IQR)	0.0 [0.0, 0.0]	0.0 [0.0, 0.0]	0.0 [0.0, 1.0]	0.0 [0.0, 0.0]	0.0 [0.0, 0.0]	0.189
NIHSS score at admission, median (IQR)	3.0 [1.0, 6.0]	4.0 [1.0, 7.0]	3.0 [1.0, 6.0]	3.0 [1.0, 5.0]	3.0 [1.0, 5.0]	<0.001
TOAST classification, n (%)						<0.001
Large-artery atherosclerosis	2,370 (24.9)	595 (26.5)	665 (27.1)	587 (24.2)	523 (21.7)	
Cardioembolism	588 (6.2)	146 (6.5)	136 (5.5)	157 (6.5)	149 (6.2)	
Small-vessel occlusion	2043 (21.4)	409 (18.2)	537 (21.9)	542 (22.3)	555 (23.0)	
Other determined etiology	136 (1.4)	34 (1.5)	32 (1.3)	39 (1.6)	31 (1.3)	
Undetermined etiology	4,399 (46.1)	1,059 (47.2)	1,087 (44.2)	1,103 (45.4)	1,150 (47.8)	
*Medical history, n (%)*						
Hypertension	5,981 (62.7)	1,382 (61.6)	1,574 (64.1)	1,541 (63.5)	1,484 (61.6)	0.181
Diabetes mellitus	2,234 (23.4)	529 (23.6)	633 (25.8)	579 (23.8)	493 (20.5)	<0.001
Dyslipidemia	738 (7.7)	159 (7.1)	176 (7.2)	200 (8.2)	203 (8.4)	0.177
Previous stroke	2029 (21.3)	402 (17.9)	550 (22.4)	542 (22.3)	535 (22.2)	<0.001
Coronary heart disease	930 (9.8)	241 (10.7)	228 (9.3)	230 (9.5)	231 (9.6)	0.332
*Medication in hospital, n (%)*						
Antiplatelet agents	9,218 (96.7)	2,152 (95.9)	2,384 (97.1)	2,351 (96.8)	2,331 (96.8)	0.156
Anticoagulant agents	948 (9.9)	289 (12.9)	245 (10.0)	221 (9.1)	193 (8.0)	<0.001
Lipid-lowering agents	9,214 (96.6)	2,160 (96.3)	2,377 (96.8)	2,339 (96.3)	2,338 (97.1)	0.366
*Laboratory tests*						
TC (mmol/L), mean (SD)	4.3 (1.2)	4.4 (1.3)	4.3 (1.2)	4.2 (1.2)	4.2 (1.2)	<0.001
HDL-C (mmol/L), mean (SD)	1.1 (0.3)	1.2 (0.3)	1.1 (0.3)	1.1 (0.3)	1.1 (0.3)	0.002
LDL-C (mmol/L), mean (SD)	2.6 (1.0)	2.7 (1.1)	2.6 (1.0)	2.5 (1.0)	2.5 (1.0)	<0.001
TG (mmol/L), median (IQR)	1.4 [1.0, 1.9]	1.4 [1.0, 1.9]	1.4 [1.0, 2.0]	1.4 [1.0, 1.9]	1.4 [1.0, 1.9]	0.147
ALT (U/L), median (IQR)	18.0 [13.0, 25.0]	17.0 [12.3, 24.0]	18.0 [13.0, 25.0]	18.0 [13.0, 26.0]	18.0 [13.0, 26.0]	<0.001
AST (U/L), median (IQR)	19.0 [16.0, 24.0]	18.7 [15.0, 23.0]	19.0 [16.0, 23.4]	19.9 [16.0, 24.0]	20.0 [16.0, 26.0]	<0.001
eGFR (mL/min/1.73 m^2^), median (IQR)	93.0 [81.3, 101.8]	93.6 [81.2, 102.4]	93.6 [82.5, 102.5]	92.8 [81.0, 101.7]	92.0 [80.2, 100.7]	<0.001
hs-CRP (mg/L), median (IQR)	1.8 [0.8, 4.7]	2.1 [0.9, 5.8]	1.8 [0.8, 4.4]	1.7 [0.8, 4.3]	1.7 [0.8, 4.3]	<0.001

Continuous variables are expressed as the median (interquartile range). Categorical variables are expressed as the frequency (percentage). ALT, alanine aminotransferase; AST, aspartate aminotransferase; eGFR, estimated glomerular filtration rate; HDL-C, high-density lipoprotein cholesterol; hs-CRP, high sensitivity C-reactive protein; LDL, low-density lipoprotein cholesterol; mRS, modified Rankin Scale; NIHSS, National Institutes of Health Stroke Scale; TC, total cholesterol; TG, triglycerides; TOAST, Trial of ORG 10172 in Acute Stroke Treatment.

### Functional outcome

**Figure 1:**
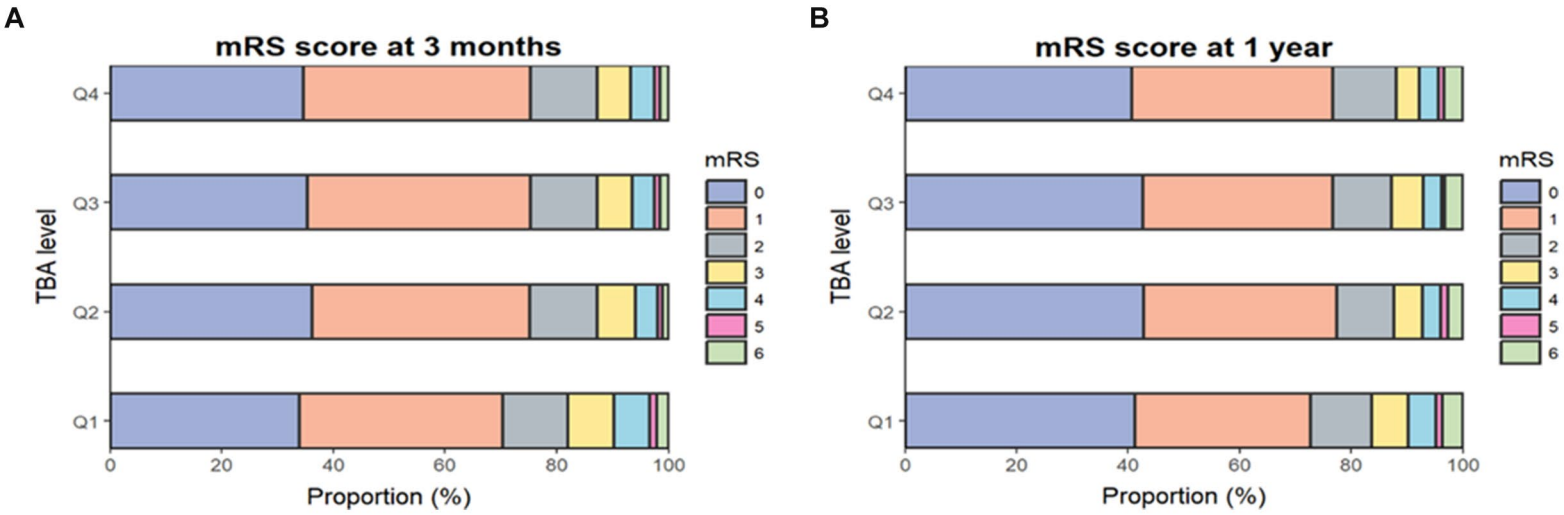
Proportions of mRS scores by TBA quartile. Distribution of mRS scores at 3 months **(A)** and 1 year **(B)**. The mRS score ranges from 0 to 6 (0, no symptoms; 1, no clinical disability; 2, slight disability; 3, moderate disability; 4, moderately severe disability; 5, severe disability; 6, death). mRS, modified Rankin Scale; TBA, total bile acids.

At the 3-month follow-up, 1,189 patients (12.7%) had an mRS score of 3–5 and 1,335 (14%) had an mRS score of 3–6 (Figure 1). After adjusting for covariates (model 3), the risks of disability and poor functional outcomes were significantly lower in the highest quartile than in the lowest quartile, with respective ORs of 0.65 (95% CI 0.55–0.78; *p* < 0.001) and 0.66 (95% CI 0.55–0.78; *p* < 0.001). Each SD increase in TBA reduced the risks of disability and poor functioning outcomes by 10% (adjusted ORs 0.9 [95% CI 0.83–0.98; *p* = 0.01] and 0.9 [95% CI 0.83–0.97; *p* < 0.001], respectively) (Table 2).

**Table 2:** Associations of total bile acids with disability and poor functional outcomes.

	Events, n (%)	Unadjusted	*p*	Model 1	*p*	Model 2	*p*	Model 3	*p*
*At 3 months*									
mRS score 3–5									
Per SD increase	1,189 (12.7)	0.9 (0.83, 0.98)	0.012	0.87 (0.8, 0.95)	0.001	0.89 (0.82, 0.96)	0.005	0.9 (0.83, 0.98)	0.010
Q1	357 (16.3)	Reference		Reference		Reference		Reference	
Q2	288 (11.9)	0.69 (0.59, 0.82)	<0.001	0.69 (0.58, 0.82)	<0.001	0.67 (0.56, 0.79)	<0.001	0.68 (0.58, 0.82)	<0.001
Q3	273 (11.4)	0.66 (0.56, 0.79)	<0.001	0.64 (0.54, 0.76)	<0.001	0.64 (0.54, 0.76)	<0.001	0.67 (0.56, 0.79)	<0.001
Q4	271 (11.4)	0.66 (0.56, 0.79)	<0.001	0.61 (0.51, 0.72)	<0.001	0.63 (0.53, 0.75)	<0.001	0.65 (0.55, 0.78)	<0.001
mRS score 3–6									
Per SD increase	1,335 (14.0)	0.90 (0.83, 0.97)	0.007	0.86 (0.80, 0.94)	<0.001	0.88 (0.82, 0.96)	0.002	0.90 (0.83, 0.97)	0.006
Q1	403 (18.0)	Reference		Reference		Reference		Reference	
Q2	314 (12.8)	0.67 (0.57, 0.78)	<0.001	0.67 (0.57, 0.79)	<0.001	0.65 (0.55, 0.77)	<0.001	0.67 (0.57, 0.79)	<0.001
Q3	311 (12.8)	0.67 (0.57, 0.79)	<0.001	0.64 (0.55, 0.76)	<0.001	0.64 (0.54, 0.76)	<0.001	0.67 (0.57, 0.80)	<0.001
Q4	307 (12.8)	0.67 (0.57, 0.78)	<0.001	0.61 (0.51, 0.72)	<0.001	0.63 (0.53, 0.75)	<0.001	0.66 (0.55, 0.78)	<0.001
*At 1 year*									
mRS score 3–5									
Per SD increase	963 (10.4)	0.89 (0.82, 0.98)	0.015	0.84 (0.77, 0.93)	0.001	0.86 (0.78, 0.94)	0.002	0.87 (0.79, 0.95)	0.003
Q1	284 (13.1)	Reference		Reference		Reference		Reference	
Q2	237 (9.9)	0.73 (0.61, 0.87)	0.001	0.71 (0.59, 0.86)	<0.001	0.69 (0.57, 0.83)	<0.001	0.71 (0.58, 0.85)	<0.001
Q3	233 (9.9)	0.73 (0.61, 0.87)	0.001	0.68 (0.57, 0.82)	<0.001	0.68 (0.56, 0.82)	<0.001	0.70 (0.58, 0.85)	<0.001
Q4	209 (9.0)	0.65 (0.54, 0.79)	<0.001	0.57 (0.47, 0.69)	<0.001	0.58 (0.48, 0.71)	<0.001	0.60 (0.49, 0.73)	<0.001
mRS score 3–6									
Per SD increase	1,267 (13.3)	0.91 (0.84, 0.98)	0.016	0.85 (0.79, 0.93)	<0.001	0.87 (0.8, 0.94)	0.001	0.88 (0.81, 0.96)	0.002
Q1	365 (16.3)	Reference		Reference		Reference		Reference	
Q2	303 (12.3)	0.72 (0.61, 0.85)	<0.001	0.71 (0.60, 0.85)	<0.001	0.69 (0.58, 0.82)	<0.001	0.72 (0.60, 0.85)	<0.001
Q3	311 (12.8)	0.76 (0.64, 0.89)	0.001	0.70 (0.59, 0.83)	<0.001	0.69 (0.58, 0.82)	<0.001	0.73 (0.61, 0.87)	<0.001
Q4	288 (12.0)	0.70 (0.59, 0.83)	<0.001	0.60 (0.51, 0.72)	<0.001	0.62 (0.52, 0.74)	<0.001	0.64 (0.54, 0.77)	<0.001

Odds ratios were used for an mRS score of 3–5 and an mRS score of 3–6. Model 1 was adjusted for age and sex. Model 2 was further adjusted for body mass index, current smoking status, alcohol consumption, pre-stroke mRS score, TOAST classification, and hypertension, diabetes mellitus, dyslipidemia, previous stroke, and coronary heart disease. Model 3 was adjusted further for antiplatelet agents, anticoagulant agents, estimated glomerular filtration rate, and high sensitivity C-reactive protein. mRS, modified Rankin Scale. SD, standard deviation; TOAST, Trial of ORG 10172 in Acute Stroke Treatment.

**Figure 2:**
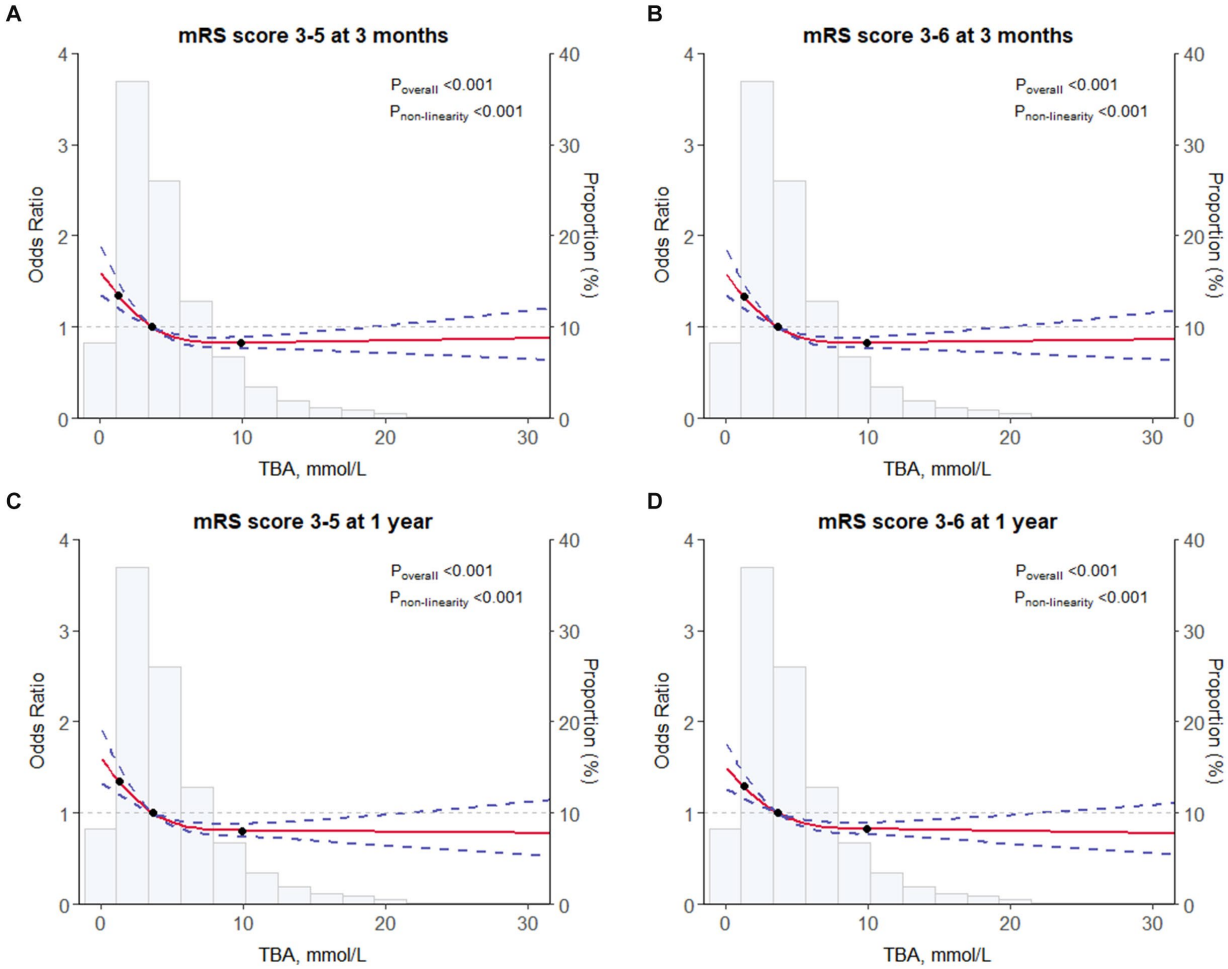
Restricted cubic spline for associations between TBA and clinical outcomes. **(A)** mRS score of 3–5 at 3 months. **(B)** mRS score of 3–6 at 3 months. **(C)** mRS score of 3–5 at 1 year. **(D)** mRS score of 3–6 at 1 year. The odds ratios from the logistic regression model were adjusted for the variables in model 3 in Table 2. mRS, modified Rankin Scale; TBA, total bile acids.

At the 1-year assessment, 963 patients (10.4%) had an mRS score of 3–5 and 1,267 (13.3%) had an mRS score of 3–6 (Figure 1). In the fully adjusted model (model 3), patients in the highest quartile had a 40% lower risk of disability and a 36% lower risk of a poor functional outcome when compared with those in the lowest quartile, with respective ORs of 0.6 (95% CI 0.49–0.73; *p* < 0.003) and 0.64 (95% CI 0.54–0.77; *p* < 0.001). Each SD increase in TBA reduced the risks of disability and poor functional outcomes by 13 and 12%, respectively (adjusted ORs 0.87 [95% CI 0.79–0.95; *p* = 0.003] and 0.88 [95% CI 0.81–0.96; *p* = 0.002]) (Table 2). Furthermore, restricted cubic spline analysis revealed a J-shaped association of serum TBA with disability and poor functional outcomes at 3 months and 1 year (Figures 2A–D).

### TOAST classification

**Figure 3:**
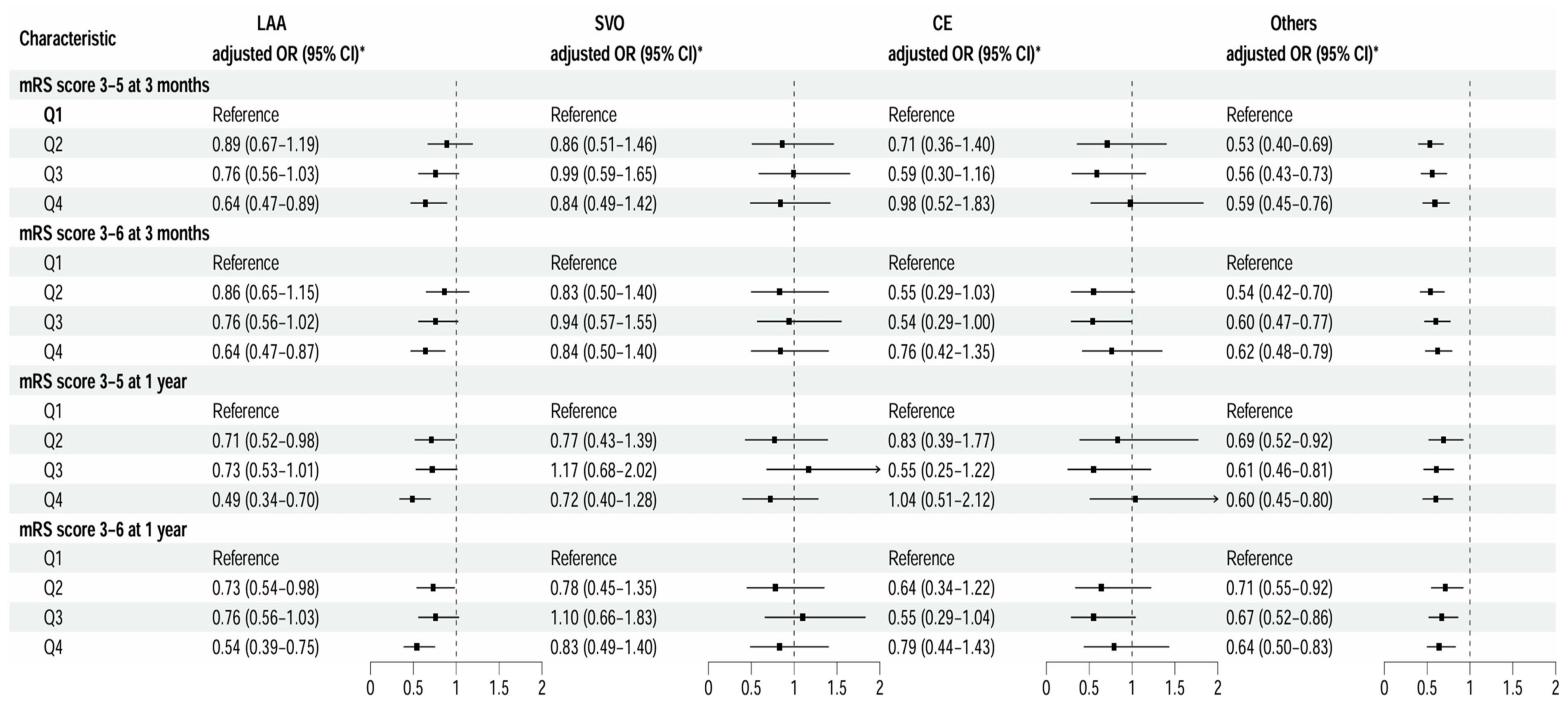
Multivariable analysis of disability and poor functional outcomes according to TOAST classification. *Model 3 is adjusted for age, sex, body mass index, current smoking status, alcohol consumption status, pre-stroke mRS score, TOAST classification, hypertension, diabetes, dyslipidemia, coronary heart disease, and previous stroke, antiplatelet agents, anticoagulant agents, estimated glomerular filtration rate, and high sensitivity C-reactive protein. CE, cardioembolism; LAA, large-artery atherosclerosis; mRS, modified Rankin Scale; Others, other determined etiology and undetermined etiology; SVO, small-vessel occlusion; TOAST, Trial of ORG 10172 in Acute Stroke Treatment.

At the 3-month follow-up, after adjusting for covariates (model 3), patients in the highest LAA subtype quartile had significantly reduced risks of disability and poor functional outcomes compared to those in the lowest quartile, with respective ORs of 0.64 (95% CI 0.47–0.89; *p* = 0.008) and 0.64 (95% CI 0.47–0.87; *p* = 0.005) (Figure 3). Furthermore, each SD increase in TBA was associated with a 17% reduction in the risk of disability and poor functional outcomes (adjusted ORs 0.83 [95% CI 0.71–0.98; *p* = 0.025] and 0.83 [95% CI 0.71–0.97; *p* = 0.018], respectively) (Supplementary Table S2). The results were similar at the 1-year (Supplementary Table S2).

At the 3-month follow-up, compared with the lowest quartile, patients in the highest quartile for the “Other” subtype had a 41% lower risk of disability and a 38% lower risk of poor functional outcomes (adjusted ORs 0.59 [95% CI 0.45–0.76; *p* < 0.001] and 0.62 [95% CI 0.48–0.79; *p* < 0.001], respectively) (Figure 3). This association was also observed at the 1-year. At the 3-month follow-up, each SD increase in TBA was associated with a 16% reduction in the risk of disability and a 15% reduction in the risk of an adverse functional outcome (adjusted ORs 0.84 [95% CI 0.72–0.96; *p* = 0.013] and 0.85 [95% CI 0.74–0.97; *p* = 0.014]). However, there was no statistically significant association at the 1-year (*p* > 0.05). Furthermore, there was no association of TBA with the small-vessel occlusion or cardioembolism subtype (Supplementary Table S2).

## Discussion

The results of this study suggest that serum TBA is an independent prognostic factor for severe disability and poor functional outcomes after AIS or TIA. Higher TBA levels were associated with decreased risks of disability and poor functional outcomes in patients with ischemic stroke. This association was also observed in the LAA and Undetermined etiology subtypes of the TOAST classification, providing a foundation for future precision treatments.

There have been limited studies investigating the association between BA levels and stroke. Animal studies have demonstrated that overexpression of the farnesoid X receptor in BAs can protect mice from myocardial infarction, exerting antiapoptotic, angiogenic, and antifibrotic effects (Xia et al., 2022). Furthermore, activation of the BA receptor TGR5 inhibits neuroinflammation and reduces brain damage (Liang et al., 2021). The endogenous BA (TUDCA) has been shown to reduce brain injury and improve neurological function in rats after intracerebral hemorrhage (Rodrigues et al., 2003). A prospective follow-up study revealed that average BA excretion was significantly higher in patients without stroke than in patients with stroke. Patients with lower excretion had higher rates of stroke and mortality, indicating that reduced BA excretion was an independent risk factor for stroke (Charach et al., 2020). A study of 777 patients with AIS found that an elevated TBA level reduced 3-month mortality (Huang L. L. et al., 2022). Furthermore, traumatic brain injury was shown to cause gastrointestinal dysfunction in patients and significantly reduction of BA levels (Zhu et al., 2021). Our study also found that the higher the BA level in patients with ischemic stroke, the less the impact on neurological function and the better the long-term recovery. Ischemic stroke can cause metabolic disturbances in the gut microbiota, particularly in the metabolism of BAs (Cai J. W. et al., 2022; Honarpisheh et al., 2022; Clottes et al., 2023). An increasing body of research suggests that metabolites derived from the gut microbiota can be considered biomarkers and mediators of stroke (Lee et al., 2020; Xu et al., 2022; Zhang et al., 2022). Therefore, BAs may have potential therapeutic value in the recovery of neurological function in patients with ischemic stroke (Figure 3).

Previous research has indicated that the gut microbiota can independently promote development of significant risk factors for stroke, including hypertension, diabetes, coronary heart disease, and atherosclerotic plaques (Durgan et al., 2019; Witkowski et al., 2020; Honarpisheh et al., 2022). BAs are excreted in feces as metabolites of cholesterol in the form of bile salts and reduce the formation of atherosclerosis by regulating lipid metabolism and activating different receptor pathways (Huang L. L. et al., 2022). Our study found a significant association of TBA with the LAA and undetermined etiological subtypes of ischemic stroke. TBA was identified as an independent risk factor for adverse functional outcomes and disability, and there was an inverse relationship between the two. The group with the lowest TBA values had a higher proportion of patients with coronary heart disease, while the undetermined etiology subtype was primarily associated with various risk factors for cardiovascular disease and atherosclerotic plaque (Ntaios, 2020). From these findings, it can be inferred that the intestinal microbial metabolites BA impacts the prognosis by promoting the mechanism of atherosclerosis.

Ischemic stroke can induce rapid gut microbiota dysbiosis, leading to gastrointestinal complications in up to 50% of patients (Xu et al., 2021; Honarpisheh et al., 2022). After dysbiosis, BAs derived from bacterial flora also appear to be abnormal. Furthermore, BAs play a dual role in regulating gut dysbiosis and participation in the gut-brain axis regulatory pathway, which is primarily achieved through changes in the intestinal immune system, inflammation and endocrine substances (Martire et al., 1988; Mertens et al., 2017; Arya and Hu, 2018; Cai J. et al., 2022; Singh et al., 2022). Our study found that high BA levels can promote recovery of neurological function, which may be regulated through the gut-brain axis pathway. In a rodent middle cerebral artery occlusion model, bile acid sequestrants confered metabolic benefits and neuroprotective effects in obesity, perhaps by modulating gut microbial composition and BA metabolism (Liang et al., 2023). Xu et al. (2021) have reported that dysbiosis of the gut microbiota accelerates deterioration of post-stroke cerebral infarction mainly through inflammation, and BA is actively involved in the intestinal inflammation process (Singh et al., 2016; Sinha et al., 2020; Honarpisheh et al., 2022; Hu et al., 2022; Clottes et al., 2023; Yin et al., 2023). Interestingly, this study also found a decreasing trend in the level of the inflammatory marker hs-CRP from the lowest BA quartile to the highest quartile, suggesting a potential association of hs-CRP with the ability of BAs to attenuate the inflammatory response. A similar phenomenon was found in the study by Huang L. L. et al. (2022). Therefore, administration of a BA supplement after stroke may become a potential novel therapeutic target in translational medicine and requires further exploration.

The study’s advantage lies in its multicenter, large-scale, prospective design, enhancing result generalizability. It is the first to investigate the relationship of TBA with disability and poor functional outcomes in AIS or TIA patients using an etiological analysis, providing insight into potential stroke treatment involving microbiota metabolite BA. However, limitations exist. While the multivariable adjustment model controlled for several important potential confounding factors, some factors such as dietary patterns, lifestyle and antibiotic use were not fully eliminated, potentially impacting the secretion of BAs (Vrieze et al., 2014; Schoeler and Caesar, 2019; Peh et al., 2022). Furthermore, patients with a history of obstructive biliary disease were not excluded due to the unavailability of such data in the CNSR-III database. The lack of examination of different types of BA makes it difficult to assess the relationship between each type of BA and clinical outcomes after stroke. Despite efforts were made to minimize bias through standard laboratory testing according to the CNSR III technical manual, heterogeneity in equipment may still introduce bias. Our study only examined TBA levels in the acute phase was lacking continuity. Therefore, it remains unclear whether changes in TBA levels may impact in turn the outcomes of IS. Additionally, as all study participants were Chinese, generalizability to other races and ethnicities may be limited.

## Conclusion

Serum BAs were found to be an independent risk factor for disability and poor functional outcomes after AIS or TIA. Higher TBA levels were associated with a reduced risk of disability and adverse functional outcomes in patients with the LAA and “other” subtypes. These findings provide a foundation for precision treatment strategies in the future.

## Data availability statement

The original contributions presented in the study are included in the article/Supplementary material, further inquiries can be directed to the corresponding authors.

## Ethics statement

The studies involving humans were approved by Beijing Tiantan Hospital (approval number KY2015-001-01). The studies were conducted in accordance with the local legislation and institutional requirements. The participants provided their written informed consent to participate in this study. Written informed consent was obtained from the individual(s) for the publication of any potentially identifiable images or data included in this article.

## Author contributions

ZW: Conceptualization, Investigation, Writing – original draft, Writing – review & editing. JGL: Data curation, Formal analysis, Writing – original draft. YX: Investigation, Supervision, Writing – original draft. YL: Data curation, Investigation, Writing – original draft. ZZ: Formal analysis, Investigation, Software, Writing – review & editing. QX: Data curation, Formal analysis, Methodology, Writing – review & editing. JXL: Data curation, Methodology, Writing – review & editing. YJ: Data curation, Methodology, Writing – review & editing. YW: Conceptualization, Investigation, Resources, Writing – review & editing. JJ: Conceptualization, Investigation, Project administration, Supervision, Writing – review & editing. AW: Data curation, Methodology, Project administration, Supervision, Writing – review & editing. XM: Conceptualization, Investigation, Project administration, Resources, Supervision, Writing – review & editing.
