# Supplementary material for: Elevated gut microbiota metabolite bile acids confer protective effects on clinical prognosis in ischemic stroke patients
Source: Front Neurosci. 2024 Apr 8;18:1388748. doi: 10.3389/fnins.2024.1388748 (PMC11033300; doi:10.3389/fnins.2024.1388748)
Supplement: Supplementary file 1 [file Data_Sheet_1.docx]

**SUPPLEMENTAL MATERIAL**


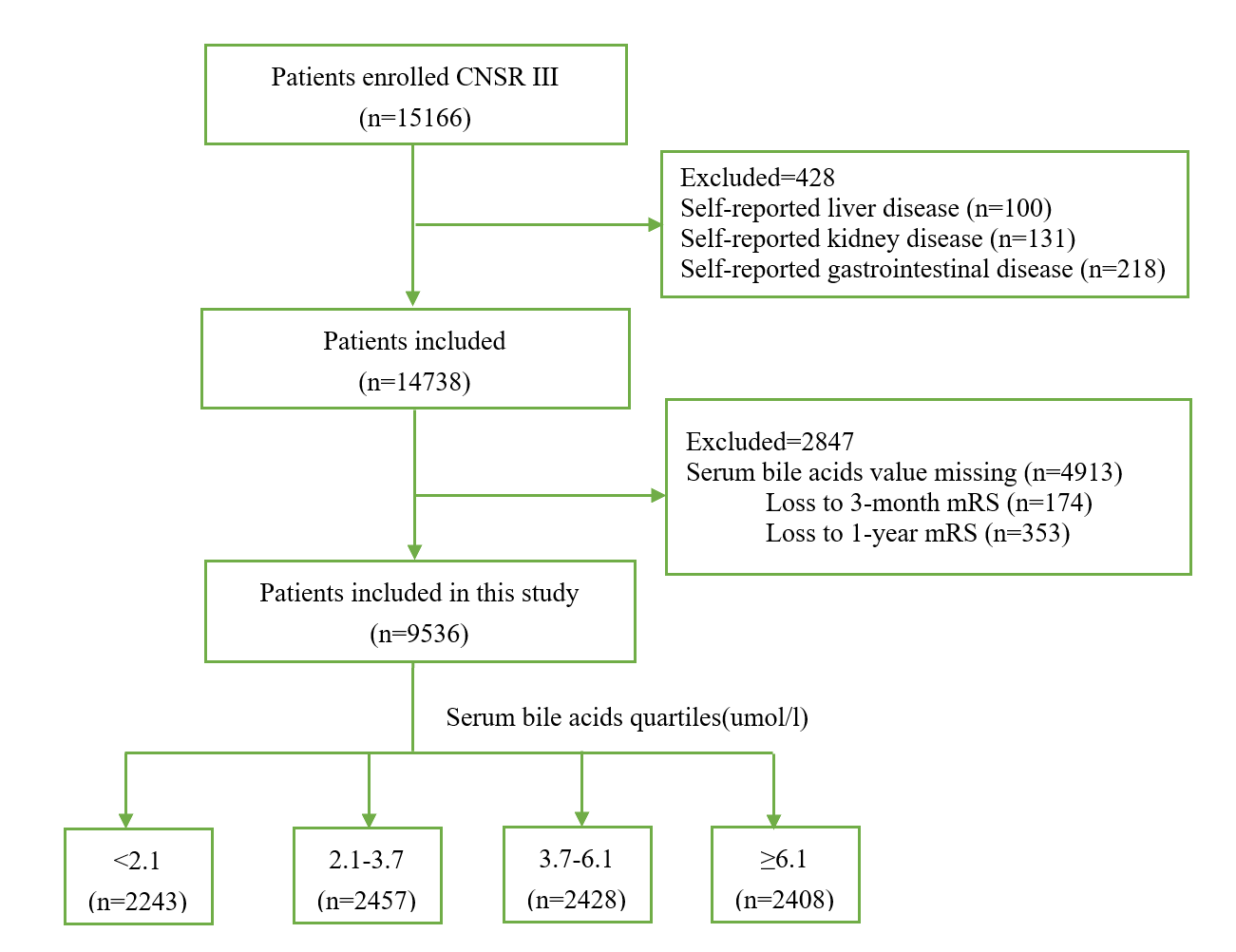


Figure S1. Patient flow diagram. CNSR-III, Third China National Stroke Registry

Table S1. Comparison between participants who were included and those who were excluded

|  | | Overall (n=15166) | Excluded (n=3070) | Included (n=12096) | P |
| --- | --- | --- | --- | --- | --- |
| n | 15166 | | 5630 | 9536 |  |
| Age, years | 63.0 [54.0, 70.0] | | 63.0 [54.0, 70.0] | 62.0 [54.0, 70.0] | 0.965 |
| Men, n (%) | 10364 (68.3) | | 3895 (69.2) | 6469 (67.8) | 0.089 |
| Body mass index, kg/m^2^ | 24.5 [22.6, 26.6] | | 24.5 [22.9, 26.6] | 24.5 [22.5, 26.6] | 0.001 |
| Current smoking, n (%) | 4752 (31.3) | | 1707 (30.3) | 3045 (31.9) | 0.040 |
| Heavy drinking, n (%) | 2126 (14.0) | | 779 (13.8) | 1347 (14.1) | 0.638 |
| Prestroke mRS score | 0.0 [0.0, 1.0] | | 0.0 [0.0, 1.0] | 0.0 [0.0, 0.0] | <0.001 |
| NIHSS score at admission | 3.0 [1.0, 6.0] | | 3.0 [1.0, 6.0] | 3.0 [1.0, 6.0] | 0.001 |
| TOAST classification, n (%) |  | |  |  | 0.001 |
| Large-artery atherosclerosis | 3856 (25.4) | | 1486 (26.4) | 2370 (24.9) |  |
| Cardioembolism | 917 ( 6.0) | | 329 ( 5.8) | 588 ( 6.2) |  |
| Small-vessel occlusion | 3165 (20.9) | | 1122 (19.9) | 2043 (21.4) |  |
| Other determined etiology | 182 ( 1.2) | | 46 ( 0.8) | 136 ( 1.4) |  |
| Undetermined etiology | 7046 (46.5) | | 2647 (47.0) | 4399 (46.1) |  |
| Medical history, n (%) |  | |  |  |  |
| Hypertension | 9494 (62.6) | | 3513 (62.4) | 5981 (62.7) | 0.705 |
| Diabetes mellitus | 3510 (23.1) | | 1276 (22.7) | 2234 (23.4) | 0.291 |
| Dyslipidemia | 1191 ( 7.9) | | 453 ( 8.0) | 738 ( 7.7) | 0.517 |
| Previous ischemic stroke | 3355 (22.1) | | 1326 (23.6) | 2029 (21.3) | 0.001 |
| Coronary heart disease | 1608 (10.6) | | 678 (12.0) | 930 ( 9.8) | <0.001 |
| Medication in hospital, n (%) |  | |  |  |  |
| Antiplatelet agents | 14613 (96.4) | | 5395 (95.8) | 9218 (96.7) | 0.009 |
| Anticoagulant agents | 1546 (10.2) | | 598 (10.6) | 948 ( 9.9) | 0.190 |
| Lipid-lowering agents | 14506 (95.7) | | 5292 (94.0) | 9214 (96.6) | <0.001 |
| Laboratory tests |  | |  |  |  |
| TC, mmol/L | 4.1 [3.4, 4.9] | | 4.1 [3.4, 4.9] | 4.2 [3.4, 4.9] | 0.128 |
| HDL-C, mmol/L | 1.1 [0.9, 1.3] | | 1.1 [0.9, 1.3] | 1.1 [0.9, 1.3] | <0.001 |
| LDL-C, mmol/L | 2.4 [1.8, 3.1] | | 2.4 [1.9, 3.1] | 2.4 [1.8, 3.1] | 0.838 |
| TG, mmol/L | 1.4 [1.0, 1.9] | | 1.4 [1.0, 1.9] | 1.4 [1.0, 1.9] | 0.054 |
| ALT, U/L | 18.0 [13.0, 26.0] | | 18.0 [13.0, 26.0] | 18.0 [13.0, 25.0] | 0.016 |
| AST, U/L | 19.0 [16.0, 24.0] | | 19.0 [15.6, 24.0] | 19.0 [16.0, 24.0] | 0.064 |
| eGFR | 93.2 [81.6, 102.0] | | 93.7 [82.3, 102.5] | 93.0 [81.3, 101.8] | 0.007 |
| hsCRP | 1.8 [0.8, 4.8] | | 1.9 [0.8, 5.0] | 1.8 [0.8, 4.7] | 0.149 |

Abbreviations: ALT, alanine aminotransferase; AST, aspartate aminotransferase; eGFR, estimated glomerular filtration rate; HDL-C, high-density lipoprotein cholesterol; hs-CRP, high sensitivity C-reactive protein; LDL, low-density lipoprotein cholesterol; mRS, modified Rankin Scale; NIHSS, National Institutes of Health Stroke Scale; TC, total cholesterol; TG, triglycerides; TOAST, Trial of ORG 10172 in Acute Stroke Treatment

Table S2. Associations of total bile acids with disability and poor functional outcomes according to TOAST classification in model 3

|  | **LAA** | ***P*** | **SVO** | ***P*** | **CE** | ***P*** | **Others** | ***P*** |
| --- | --- | --- | --- | --- | --- | --- | --- | --- |
| **At 3 months** |  |  |  |  |  |  |  |  |
| mRS score 3-5 |  |  |  |  |  |  |  |  |
| Events, n (%) | 429 (4.6) |  | 139 (1.5) |  | 95 (1.0) |  | 526 (5.6) |  |
| Per SD increase | 0.83 (0.71, 0.98) | 0.025 | 0.98 (0.82, 1.18) | 0.857 | 1.09 (0.91, 1.32) | 0.337 | 0.84 (0.72, 0.96) | 0.013 |
| Q1 | Reference |  | Reference |  | Reference |  | Reference |  |
| Q2 | 0.89 (0.67, 1.19) | 0.448 | 0.86 (0.51, 1.46) | 0.586 | 0.71 (0.36, 1.40) | 0.320 | 0.53 (0.40, 0.69) | <0.001 |
| Q3 | 0.76 (0.56, 1.03) | 0.073 | 0.99 (0.59, 1.65) | 0.969 | 0.59 (0.30, 1.16) | 0.124 | 0.56 (0.43, 0.73) | <0.001 |
| Q4 | 0.64 (0.47, 0.89) | 0.008 | 0.84 (0.49, 1.42) | 0.507 | 0.98 (0.52, 1.83) | 0.944 | 0.59 (0.45, 0.76) | <0.001 |
| mRS score 3-6 |  |  |  |  |  |  |  |  |
| Events, n (%) | 473 (5.0) |  | 145 (1.5) |  | 116 (1.2) |  | 601 (6.3) |  |
| Per SD increase | 0.83 (0.71, 0.97) | 0.018 | 1.01 (0.85, 1.19) | 0.938 | 1.02 (0.85, 1.23) | 0.821 | 0.85 (0.74, 0.97) | 0.014 |
| Q1 | Reference |  | Reference |  | Reference |  | Reference |  |
| Q2 | 0.86 (0.65, 1.15) | 0.312 | 0.83 (0.50, 1.40) | 0.488 | 0.55 (0.29, 1.03) | 0.064 | 0.54 (0.42, 0.70) | <0.001 |
| Q3 | 0.76 (0.56, 1.02) | 0.065 | 0.94 (0.57, 1.55) | 0.821 | 0.54 (0.29, 1.00) | 0.049 | 0.60 (0.47, 0.77) | <0.001 |
| Q4 | 0.64 (0.47, 0.87) | 0.005 | 0.84 (0.50, 1.40) | 0.500 | 0.76 (0.42, 1.35) | 0.341 | 0.62 (0.48, 0.79) | <0.001 |
| **At 1 year** |  |  |  |  |  |  |  |  |
| mRS score 3-5 |  |  |  |  |  |  |  |  |
| Events, n (%) | 339 (3.7) |  | 117 (1.3) |  | 73 (0.8) |  | 434 (4.7) |  |
| Per SD increase | 0.72 (0.59, 0.89) | 0.002 | 0.95 (0.77, 1.18) | 0.674 | 1.05 (0.84, 1.31) | 0.681 | 0.87 (0.75, 1.01) | 0.069 |
| Q1 | Reference |  | Reference |  | Reference |  | Reference |  |
| Q2 | 0.71 (0.52, 0.98) | 0.036 | 0.77 (0.43, 1.39) | 0.381 | 0.83 (0.39, 1.77) | 0.622 | 0.69 (0.52, 0.92) | 0.010 |
| Q3 | 0.73 (0.53, 1.01) | 0.058 | 1.17 (0.68, 2.02) | 0.574 | 0.55 (0.25, 1.22) | 0.141 | 0.61 (0.46, 0.81) | 0.001 |
| Q4 | 0.49 (0.34, 0.70) | <0.001 | 0.72 (0.40, 1.28) | 0.262 | 1.04 (0.51, 2.12) | 0.907 | 0.60 (0.45, 0.80) | <0.001 |
| mRS score 3-6 |  |  |  |  |  |  |  |  |
| Events, n (%) | 432 (4.5) |  | 140 (1.5) |  | 115 (1.2) |  | 580 (6.1) |  |
| Per SD increase | 0.76 (0.64, 0.91) | 0.003 | 1.03 (0.88, 1.21) | 0.681 | 0.93 (0.75, 1.15) | 0.482 | 0.88 (0.77, 1.00) | 0.053 |
| Q1 | Reference |  | Reference |  | Reference |  | Reference |  |
| Q2 | 0.73 (0.54, 0.98) | 0.034 | 0.78 (0.45, 1.35) | 0.369 | 0.64 (0.34, 1.22) | 0.179 | 0.71 (0.55, 0.92) | 0.009 |
| Q3 | 0.76 (0.56, 1.03) | 0.080 | 1.10 (0.66, 1.83) | 0.719 | 0.55 (0.29, 1.04) | 0.067 | 0.67 (0.52, 0.86) | 0.002 |
| Q4 | 0.54 (0.39, 0.75) | <0.001 | 0.83 (0.49, 1.40) | 0.481 | 0.79 (0.44, 1.43) | 0.437 | 0.64 (0.50, 0.83) | 0.001 |

Note: Odds ratios were used for an mRS score of 3–5 and an mRS score of 3–6. Model 3 was adjusted for age, sex, body mass index, current smoking, alcohol consumption, pre-stroke mRS score, TOAST classification, hypertension, diabetes, dyslipidemia, coronary heart disease, and previous stroke, antiplatelet agents, anticoagulant agents, estimated glomerular filtration rate, and high-sensitivity C-reactive protein. Abbreviations: CE, cardioembolism; mRS, modified Rankin Scale; LAA, large-artery atherosclerosis; SVO, small-vessel occlusion; TOAST, Trial of ORG 10172 in Acute Stroke Treatment

Table S3. Associations of total bile acids with disability and poor functional outcomes according to CCS classification in model 3

|  | **LAA** | ***P*** | **SVO** | ***P*** | **CE** | ***P*** | **Others** | ***P*** |
| --- | --- | --- | --- | --- | --- | --- | --- | --- |
| **At 3 months** |  |  |  |  |  |  |  |  |
| mRS score 3-5 |  |  |  |  |  |  |  |  |
| Per SD increase | 0.78 (0.66, 0.92) | 0.003 | 0.96 (0.81, 1.13) | 0.597 | 1.03 (0.85, 1.26) | 0.738 | 0.93 (0.81, 1.08) | 0.361 |
| Q1 | Reference |  | Reference |  | Reference |  | Reference |  |
| Q2 | 0.74 (0.56, 0.96) | 0.024 | 0.67 (0.44, 1.02) | 0.064 | 0.46 (0.24, 0.88) | 0.019 | 0.67 (0.49, 0.92) | 0.013 |
| Q3 | 0.71 (0.53, 0.94) | 0.017 | 0.84 (0.56, 1.27) | 0.410 | 0.50 (0.27, 0.92) | 0.027 | 0.60 (0.44, 0.82) | 0.001 |
| Q4 | 0.53 (0.40, 0.72) | <0.001 | 0.76 (0.50, 1.15) | 0.192 | 0.56 (0.30, 1.04) | 0.066 | 0.78 (0.58, 1.05) | 0.101 |
| mRS score 3-6 |  |  |  |  |  |  |  |  |
| Per SD increase | 0.8 0(0.69, 0.93) | 0.004 | 0.97 (0.83, 1.13) | 0.696 | 0.96 (0.79, 1.17) | 0.710 | 0.93 (0.81, 1.07) | 0.292 |
| Q1 | Reference |  | Reference |  | Reference |  | Reference |  |
| Q2 | 0.72 (0.55, 0.93) | 0.012 | 0.63 (0.42, 0.96) | 0.03 | 0.45 (0.25, 0.82) | 0.009 | 0.69 (0.51, 0.93) | 0.014 |
| Q3 | 0.75 (0.58, 0.99) | 0.041 | 0.81 (0.55, 1.21) | 0.305 | 0.49 (0.28, 0.87) | 0.015 | 0.60 (0.44, 0.81) | 0.001 |
| Q4 | 0.56 (0.42, 0.74) | <0.001 | 0.75 (0.50, 1.12) | 0.16 | 0.50 (0.28, 0.89) | 0.018 | 0.79 (0.60, 1.06) | 0.113 |
| **At 1 year** |  |  |  |  |  |  |  |  |
| mRS score 3-5 |  |  |  |  |  |  |  |  |
| Per SD increase | 0.78 (0.65, 0.93) | 0.006 | 0.90 (0.73, 1.12) | 0.358 | 0.95 (0.75, 1.2) | 0.673 | 0.89 (0.76, 1.05) | 0.172 |
| Q1 | Reference |  | Reference |  | Reference |  | Reference |  |
| Q2 | 0.7 (0.52, 0.94) | 0.018 | 0.70 (0.43, 1.15) | 0.159 | 0.70 (0.35, 1.38) | 0.302 | 0.70 (0.50, 0.97) | 0.031 |
| Q3 | 0.71 (0.52, 0.97) | 0.034 | 1.12 (0.71, 1.77) | 0.620 | 0.47 (0.23, 0.96) | 0.037 | 0.57 (0.41, 0.80) | 0.001 |
| Q4 | 0.49 (0.35, 0.69) | <0.001 | 0.65 (0.40, 1.06) | 0.085 | 0.70 (0.36, 1.36) | 0.292 | 0.66 (0.48, 0.91) | 0.012 |
| mRS score 3-6 |  |  |  |  |  |  |  |  |
| Per SD increase | 0.81 (0.70, 0.95) | 0.008 | 0.97 (0.83, 1.15) | 0.761 | 0.86 (0.69, 1.07) | 0.177 | 0.90 (0.78, 1.04) | 0.162 |
| Q1 | Reference |  | Reference |  | Reference |  | Reference |  |
| Q2 | 0.73 (0.55, 0.96) | 0.023 | 0.70 (0.44, 1.1) | 0.118 | 0.73 (0.41, 1.29) | 0.279 | 0.69 (0.51, 0.94) | 0.017 |
| Q3 | 0.82 (0.62, 1.09) | 0.172 | 1.02 (0.67, 1.56) | 0.909 | 0.54 (0.30, 0.97) | 0.038 | 0.57 (0.42, 0.78) | <0.001 |
| Q4 | 0.56 (0.41, 0.76) | <0.001 | 0.70 (0.45, 1.10) | 0.121 | 0.63 (0.35, 1.11) | 0.106 | 0.71 (0.53, 0.95) | 0.021 |

Note: Odds ratios were used for an mRS score of 3–5 and an mRS score of 3–6. Model 3 was adjusted for age, sex, body mass index, current smoking, alcohol consumption, pre-stroke mRS score, TOAST classification, hypertension, diabetes, dyslipidemia, coronary heart disease, and previous stroke, antiplatelet agents, anticoagulant agents, estimated glomerular filtration rate, and high-sensitivity C-reactive protein. Abbreviations: CE, cardioembolism; mRS, modified Rankin Scale; LAA, large-artery atherosclerosis; SVO, small-vessel occlusion; TOAST, Trial of ORG 10172 in Acute Stroke Treatment
